# Allegheny County Medical Society

**Published:** 1890-10

**Authors:** W. S. Foster

**Affiliations:** President, in the Chair


### ALLEGHENY COUNTY MEDICAL SOCIETY.

SPECIAL MEETING, AUGUST 19TH, 1890.

W. S. Foster, M. D., President, in the Chair.

### EXHIBITION OF SOME URETHRAL INSTRUMENTS.

DR. STEWART: The first instrument is the urethragraph. A year ago last July I exhibited my first urethragraph. I have made a number of changes. This instrument is for the purpose of recording a diagram of the urethra, giving the exact circumference at every point. The next instrument I will show you resembles the first one. It is almost the same in principle. It is for a different purpose; it is a combined urethrameter and urethratome, so made that it will either measure the urethra or cut a stricture. This instrument is inserted into the urethra as far as you may desire, usually to the bulbous portion. If you desire to cut a stricture, you first ascertain the size of the healthy portion of the urethra, the part you do not wish to cut, then permit the blades to open to the size you desire to cut. Having done this, fasten the screw on the upper surface and withdraw the instrument. The third instrument is the urethrascope, the fourth an urethral syringe.

DR. THOMAS: I have had no experience with the doctor's instruments. I did have a little experience in the doctor's presence with his first urethragraph, at the time it was not perfected, so that my experience will not serve in my remarks at present. The principle of the instrument is doubtless correct. I have been using the urethrameter of Professor Otis, which I have considered the best in the market. The difficulty I found was this; that you had to cover the instrument with a gum tube, the gum tube always giving more or less resistance. After passing the urethrameter down as far as it would go to the bulbous portion, and then opening its arms to the full caliber of the urethra, then in withdrawing it when you would come to a stricture, of course the instrument would cease. Then in order to pass through the stricture, you had to reduce the caliber until it would cut through the stricture, but in bringing it through the stricture you could not tell where the stricture ended. You might pull your instrument anterior to the stricture. Now, that is the trouble with the Otis urethrameter. In the doctor's urethrameter, if the spring has the proper sensibility to come and go with the inequalities of the urethra, then it is a typical instrument. But the question is to me whether you can get a spring sufficiently graduated to come and go as you withdraw the instrument.

DR. STEWART: As Dr. Thomas has suggested, the spring in the instrument we used together was weak; this has been remedied.

Dr. Werder reported five laparotomies. See page 456.

DR. MACFARLANE: It is gratifying to see so many patients relieved of tumors who do well. I do not say that it occurs in the hands of all operators, but very frequently the patient is left in a condition nearly as bad as that in which she was prior to the operation, the only effect being to prolong life for a time.

DR. WERDER: I will simply state that it is very unfortunate when the patient is left in the condition spoken of by Dr. Mac

Farlane. It is very important in performing a laparotomy not to make your abdominal incision larger than necessary. A small incision only is needed, not more than three inches. Now, if this is united very carefully, and the stitches put in at proper intervals, I think hernias will not occur very often, though they cannot always be prevented. In regard to fistulæ, those are things that will often occur from septic ligatures. If you have not your ligatures absolutely aseptic, it is very likely that a fistula will follow, but with perfectly aseptic material, it should be a very rare occurrence to have a fistula.

### CASE OF TYPHOID FEVER.

DR. KEARNS: A boy twelve years old, in the third week, ceased to speak even in monosyllables, and this condition continued for about ten days. During this time there was no apparent impairment of intellect. Sitting at the bedside of the patient and telling him to put out his tongue, he did it instantly. Telling him to look toward me that I might examine his eyes, he did it instantly. The pupils of the eyes were markedly dilated. Then at the expiration of these ten days, the case assumed the very opposite condition and became loquacious; he would take up any conversation which occurred in the room and follow it up repeatedly. This condition continued day and night with some short intervals of rest for ten days, when it gradually stopped. The pulse was accelerated during this period of excitation. It was at a normal stage during the period of quietude. All this time the stomach had been in good condition. Now here are two extremes. What condition of the brain and nervous system is involved in these conditions of two extremes in the same patient and the same disease? This cerebral excitation was very difficult to control. The simple remedy which appeared to have the desired effect was calomel. I administered a grain of calomel every two hours, then when the bowels began to run off, in smaller doses. To me this was a very interesting case, and I ascribe the nervous symptoms to a complicating meningitis.

DR. THOMAS: During the month of April, I saw a patient with typhoid fever. The boy was thirteen years old. He had been sick about a week. The fever ran an ordinary course. About the twenty-first day there was defervescence, and I presumed the case was going on to convalescence. I visited the boy as long as I remained in the city, and in the meantime he would not speak a word until the day before I went away, I got him to say one word. I did not feel uneasy about him, his temperature not being above normal. He went into the hands of Dr. McNeil. On my return, I found the boy all right, and was told that in speaking to his grandmother, in whose care he was, upon his beginning to talk again, the first word he said was cracker. He said, "cracker, cracker, cracker," for three or four minutes; then he ceased calling for crackers. I looked upon it as caused by anæmia of the brain.

DR. STEWART: I remember a case where a man lost the power of the right arm. The loss was progressive, and then he had convulsions. The convulsions were in the arm affected. Subsequently they became general, and he would become unconscious. The convulsions became very frequent, several times a day. An operation was performed under the supposition of lesion in that area. The man had had syphilis. Iodide of potassium had no effect on the case. The brain was uncovered and only a localized meningitis was found. Incisions were made into the brain and nothing was found. The man ultimately recovered the perfect use of his arm, and had no more convulsions.

DR. MCKENNAN: I find that it is not all uncommon to have peculiar mental states following typhoid fever; mental weakness, and also very frequently mental exhilaration. I have seen many cases of insanity which have been traced to typhoid fever. I have never seen a case of meningitis in a child with typhoid fever. The whole weight of authority goes toward the supposition that the lesion is purely of a functional character, and that there is rarely any structural lesion, although some authorities state the possibility that there may be embolism which could only involve one artery.

DR. LANGE: No matter what cerebral symptoms we may hav in typhoid fever, there is no justification for the assumption of meningitis. No matter how violent or how peculiar are the cerebral symptoms, the assumption of meningitis is not correct, is not justified. I do not know that typhoid fever and meningitis are incompatible, but I mean to say that *post mortem* examination in cases of typhoid fever which presented most violent and most strange ataxic symptoms have so invariably proven the absence of meningitis, and of all inflammation, that such symptoms cannot be correctly assigned to meningitis or to any structural lesion, but are to be considered only as the toxic effects of the typhoid fever poison. Neither can I understand how the speech center can be affected by a meningitis without previous and greater injury to the motor areas, which being in closer apposition to the meninges than the centre of speech, would primarily, and to a greater extent, be subjected to meningeal pressure. For this reason paralysis is as common in meningitis as aphasia (barring, of course, comatose cases) is rare.

